# Prosocial behavior of wearing a mask during an epidemic: an evolutionary explanation

**DOI:** 10.1038/s41598-021-92094-2

**Published:** 2021-06-16

**Authors:** K. M. Ariful Kabir, Tori Risa, Jun Tanimoto

**Affiliations:** 1 Department of Mathematics, Bangladesh University of Engineering and Technology, Dhaka, Bangladesh; 2 Interdisciplinary Graduate School of Engineering Sciences, Kyushu University, Kasuga-koen, Kasuga-shi, Fukuoka, 816-8580 Japan; 3 Faculty of Engineering Sciences, Kyushu University, Kasuga-koen, Kasuga-shi, Fukuoka, 816-8580 Japan

**Keywords:** Computational biology and bioinformatics, Evolution, Diseases, Mathematics and computing

## Abstract

In the midst of the COVID-19 pandemic, with limited or no supplies of vaccines and treatments, people and policymakers seek easy to implement and cost-effective alternatives to combat the spread of infection during the pandemic. The practice of wearing a mask, which requires change in people’s usual behavior, may reduce disease transmission by preventing the virus spread from infectious to susceptible individuals. Wearing a mask may result in a public good game structure, where an individual does not want to wear a mask but desires that others wear it. This study develops and analyzes a new intervention game model that combines the mathematical models of epidemiology with evolutionary game theory. This approach quantifies how people use mask-wearing and related protecting behaviors that directly benefit the wearer and bring some advantage to other people during an epidemic. At each time-step, a suspected susceptible individual decides whether to wear a facemask, or not, due to a social learning process that accounts for the risk of infection and mask cost. Numerical results reveal a diverse and rich social dilemma structure that is hidden behind this mask-wearing dilemma. Our results highlight the sociological dimension of mask-wearing policy.

## Introduction

In the absence of appropriate drugs and a reliable vaccine^1,2^ against transmittable diseases, government and health authorities have implemented a series of policies and health strategies^3–7^ to slow the spread of the coronavirus. For example, to slow off disease-spreading, individuals engaged in preventative measures, including wearing masks, staying home, and keeping physical distancing. Travel restrictions^8,9^, lockdown^10^, handwashing, and quarantine strategy^6,11,12^ also emerged as precautionary measures. Compared with suppression strategies (vaccine, drug)^13–16^, mitigation strategies (wearing masks, social/physical distancing, and handwashing)^17,18^ are more frequently adopted defensive measures that can only reduce infection risk to some extent. Further, the methods of behavioral science interventions have proven their importance in studying the interplay between disease and human decision in the social dilemma aspect^19–23^. In this work, we evaluate the effect of the choice of wearing masks as a prosocial behavior on epidemic dynamics. Using evolutionary game theory (EGT)^24–26^, we model individual’s decision whether to wear a mask as depending on several reasons, including mask cost, risk of infection, and conformity effect.

Currently, during COVID-19 pandemic^27–29^, wearing a face mask is considered an individual and public control measure against the transmission of SARS-CoV-2^30,31^. Significant controversy still exists among people in different countries regarding mask-wearing policies. When deciding whether to wear a mask, individuals consider the mask’s cost and efficiency, altruistic or prosocial behavior, conformity effect, hesitancy for wearing a mask, and risk of infection. Individuals’ decisions are directly related to the self-benefit and benefit to others because this decision increases the number of mask-wearing people and reduces the risk of infection. For example, people in Japan are comfortable in wearing a face mask^32^, both for social norms and medical purposes. Although wearing a face mask may not be the only reason Japan has a relatively low infection and death rate (3,15,910 confirmed cases with 4,380 fatalities, 17 January 2021)^33^, experts state that it is a positive contributing factor. In comparison, the United States (US) is struggling with the pandemic (24.3 million cases and more than 4,05,200 deaths, 17 January 2021)^33^ because people there view wearing masks as needless, ineffective, or infringing on their civil liberties^34,35^.

To investigate a pandemic or disease incidence, a mathematical compartmental model^36^ is a ubiquitous tool in epidemiology and public health system. The SIR dynamics, where S, I, and R represent susceptible, infected, and recovered states, respectively^37^, is one of the basic epidemiological models that describes disease transmission. In addition, the model has been expanded to consider different premises and circumstances. For example, SEIR (susceptible, exposed, infected, and recovered)^38^, SVIR (susceptible, vaccinated, infected and recovered)^39^, SIR-UA (aware–unaware)^18^, metapopulation model^40^, and quarantine–isolation epidemic model^7^ that represent the epidemiology for various perspectives as a modified model of SIR have been used. Following the approach of epidemic process, we modeled the epidemic formulation in which the population is separated into two major subgroups: mask-wearing group and non-mask wearing group. In our model, individuals move from non-mask groups to mask groups (susceptible only) based on individual choice in a game-theoretical approach EGT offers a platform for explaining individual behavior in such a setting, where the options favored by individuals depend on either wearing a mask or not^41^. This framework lets us to determine how mask-wearing helps people who wear masks and those around them. In this study, we also adopted the concept of conformity pressure^42^, mask efficiency and cost, and hesitance to incur the cost of wearing a mask cost. Finally, this model introduces the idea of social efficiency deficit (SED)^43–45^ to determine the social dilemma (a gap between Nash equilibrium (NE) and social optimum (SO)) in EGT.

Several studies have presented the importance of using a face mask against contagious diseases on an epidemiological process in which only mask effectiveness/efficiency was considered without evaluating human behavior^46–50^. Stutt et al.^51^ have imposed a modeling framework onto the SEIR model to account for face mask effectiveness with and without lockdown in reducing the transmission of SARS-CoV-2. Eikenberry et al. (2020)^30^, using the mathematical epidemic model, assessed the community-based impact of asymptomatic individuals using masks. Notably, using real data from New York and Washington, they showed that face masks can meaningfully reduce community transmission. Inspired by the influenza’s pandemic potential, some studies^48–50^, which adopted a face-mask-based mathematical model, have analyzed the necessity of wearing a face mask by the general population. As introduced by Bauch et al.^26,52^ and followed up by Kabir et al.^10,39,53,54^, the general form of social learning behavioral model on EGT, a straightforward human decision mechanism to wear a mask, has received attention. Here we also consider a recent work of the voluntary quarantine strategies governed by EGT on an epidemic framework from a single process social strategy, risk perception, and viral spreading (quarantine or not depend on the perceived risk and quarantine cost)^55^. The evolution of cooperation under EGT is an important and illustrative example, which shows how a contagious disease can encourage mask use. In addition, it is plausible that conformity pressure, mask sensitivity/hesitance, and mask cost are essential in encouraging people to wear mask. To address the effect of altruistic behavior, wearing a mask may not effectively protect a person but could help avoid dispersing the virus. Thus, wearing a mask or not may evoke an analogous structure to public good games, including the vaccination game or vaccination dilemma^14^. The vaccination dilemma allows a framework that elucidates all defector's states (since all are trying to free-riding on herd immunity) at Nash equilibrium (NE) and social optimum (SO) appears on specific cooperation fraction state ensuring herd-immunity. The mask game also presents similar phenomena; the all-defectors state in NE because wearing masks compels people to bother besides cost. However, all people wearing the mask in the cooperator state might be most robust to impede disease spreading. There is a strong motivation to skip mask-wearing to avoid mask cost; thus, people try enjoying the mask benefit from other mask-wearers. To analyze behavioral encouragement in a mask-dilemma setting, this study considers two-layer $SEI^{A} I^{S} R - S_{M} E_{M} I_{M}^{A} I_{M}^{S} R_{M}$ epidemic dynamics on the EGT framework by coupling the disease-spreading model with a mask-wearing aspect and social learning decision-making model. The choice for using this approach is based on several factors, including the following: (i) both epidemic and human decision to wear a mask occurring on the same timescale (local) are implemented; (ii) cost, efficiency, and hesitance for mask cost are considered; (iii) positive and negative conformity is designed in response to the group pressure^42^; (iv) mask-wearing to reduce the spread of disease is intended. As we explained, this is an emerging social dilemma situation. Thus, wearing a mask would be socially accepted as one of the effective preintervention measures that do not deliver direct benefit to wearing individuals but helps people around them.

## Results

**Figure 1:**
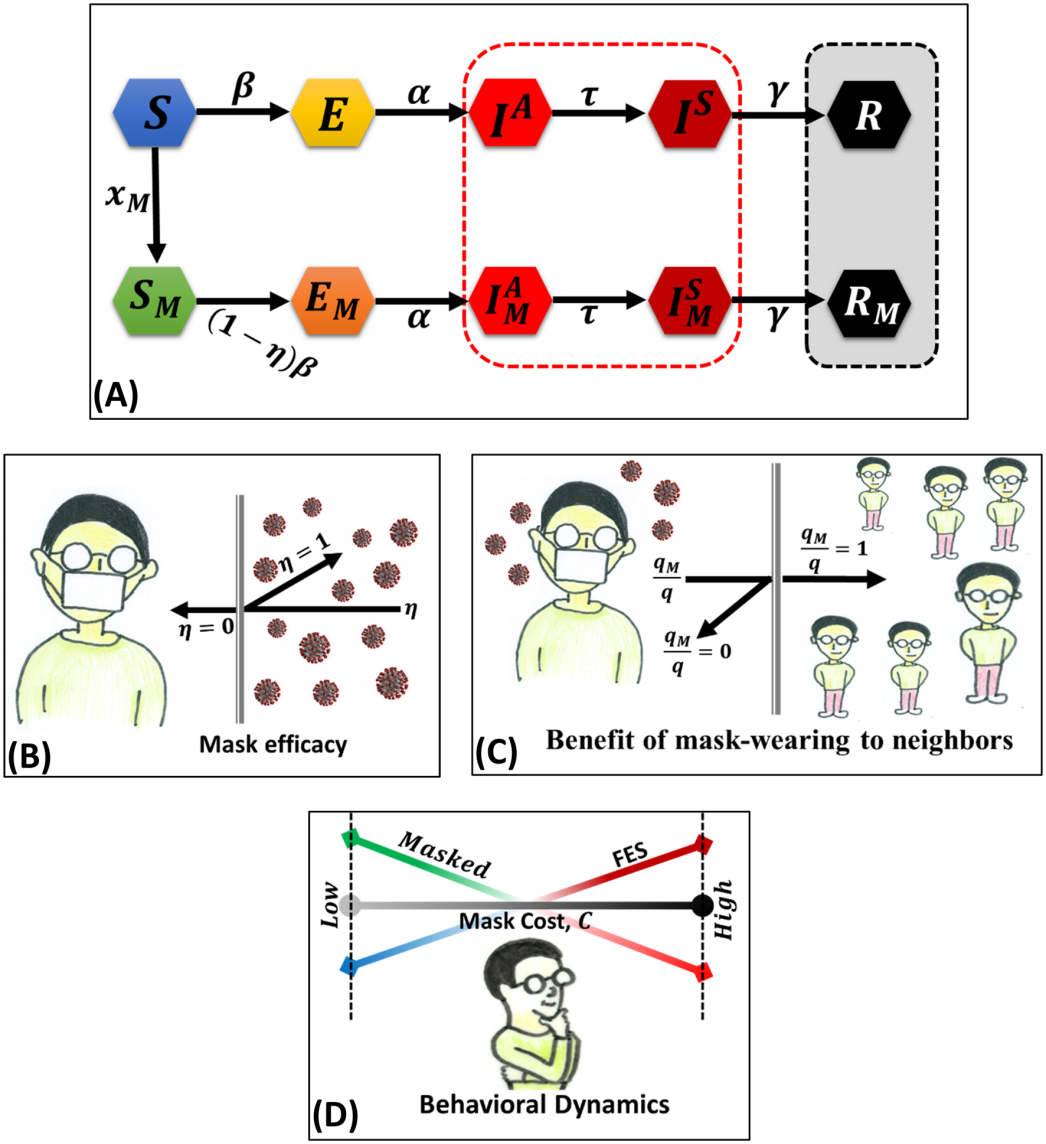
**(A)** Schematic diagram of mask-wearing and non-mask wearing individuals. The arrows that connect the compartment represent the movement of the fraction of individuals from one state to another. Non-mask wearing suspected susceptible individuals $\left( S \right)$ can either become exposed (no-mask) $\left( E \right)$ or mask-wearing susceptible $\left( {S_{M} } \right)$. Both mask- and non-mask wearing susceptible individuals $\left( {S~{\text{and~}}S_{M} } \right)$ become exposed $\left( {E~{\text{and~}}E_{M} } \right)$, asymptomatic infected $\left( {I^{A} ~{\text{and~}}I_{M}^{A} } \right)$, symptomatic infected $\left( {I^{S} ~{\text{and~}}I_{M}^{S} } \right)$, and recovered $\left( {R~{\text{and~}}R_{M} } \right)$. **(B)** The mechanism of mask efficiency $\left( \eta  \right)$ may be apparent when the effectiveness of the mask is higher (for example, N95 and KN95 mask) that protect the wearer from viruses. **(C)** While an individual is asymptomatic infected and wearing a mask, the neighbor's non-mask wearer may benefit from the mask wearer. **(D)** Apart from the disease dynamic, the evolutionary decision-making process, an individual chooses whether to wear a mask or not, somehow depends on the relative cost of the mask and perceived risk.

**Figure 2:**
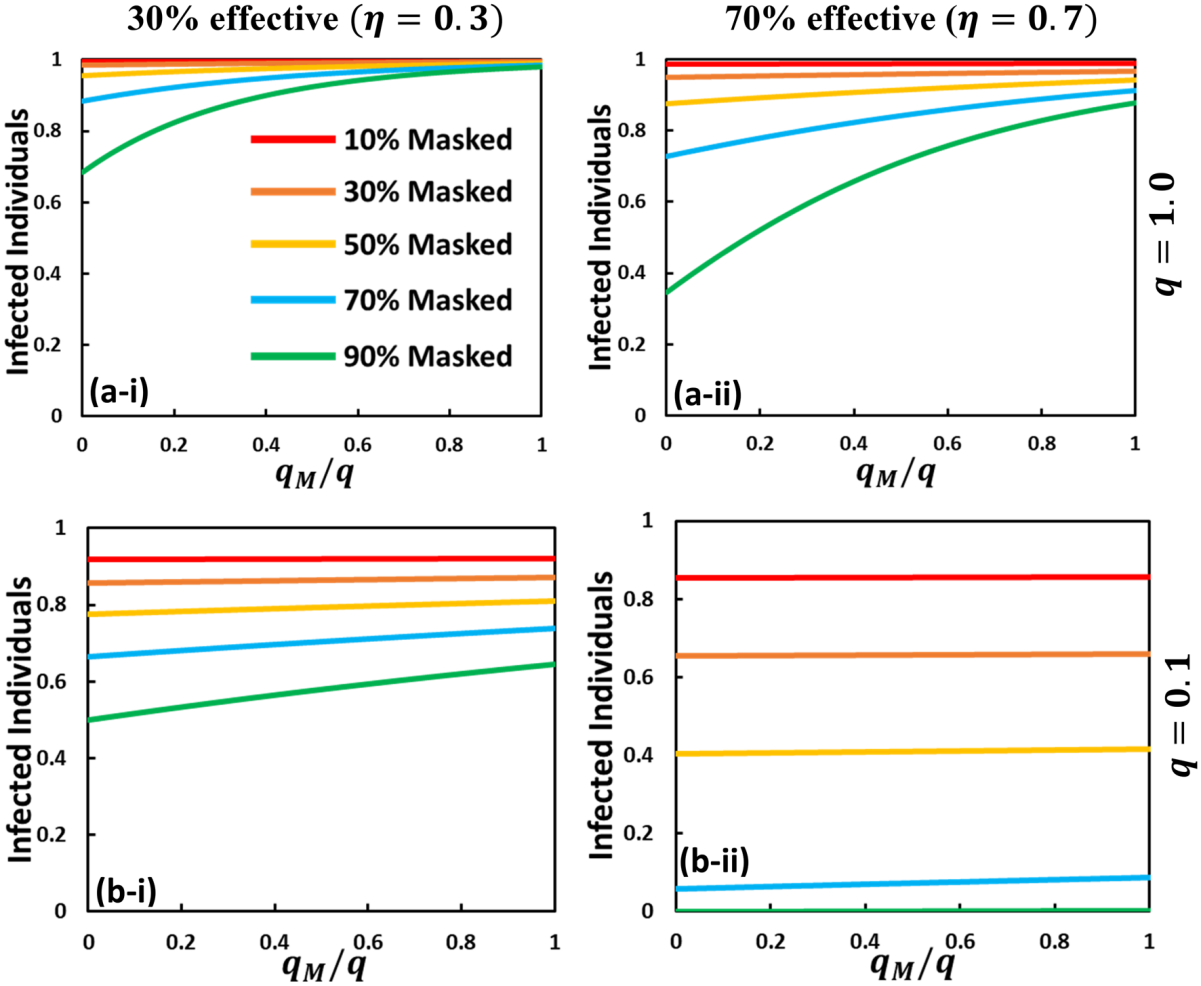
Fraction of infected individuals that is greatly affected by mask-wearing individuals if mask efficiency is high and the percentage of the population wearing a mask is reasonable. Here, the fraction of infected individuals at equilibrium along $${\raise0.7ex\hbox{${q_{M} }$} \!\mathord{\left/ {\vphantom {{q_{M} } q}}\right.\kern-\nulldelimiterspace} \!\lower0.7ex\hbox{$q$}}(0 \le q_{M}  < q)$$ is plotted for non-behavioral settings, $$x_{M} = 0.0$$. The fraction of the highest infected individuals is observed in low mask efficiency $$\left( {\eta = 0.3} \right)$$ and higher $$q\left( { = 1.0} \right)$$ at panel (a-i) [for example, clothing mask]. The infected fraction less or dies out when a joint higher effective mask (70% effective) [for example, surgical or N95 mask] and lower $$q\left( { = 1.0} \right)$$ are imposed (b-ii). For panel (a-ii) and (b-i), we choose $$\left( {\eta ,q} \right) = \left( {0.7,1.0} \right)$$ and $$\left( {0.3,0.1} \right)$$, respectively. For all four cases, the baseline parameters are presumed as, $$\beta = 0.83333, \gamma = \frac{1}{3},~\alpha = \frac{1}{6}$$, and $$\tau$$.

First, let us observe simple epidemic dynamics with a fixed 10%, 30%, 50%, 70%, and 90% of susceptible individuals wearing masks. In practice, we assume that the mask transmission rate is ${x}_{M} = 0$ and the ratio of asymptomatic to symptomatic diffusing risk without wearing a mask is $q = 1.0$ (Figs. 1, 2(a)) and $q~ = 0.1({\text{Fig}}.~2({\text{a}}))$ (where, $0\le {q}_{M}\le q)$. The numerical results of infected individuals at equilibrium with different mask efficiency ($\eta = 0.3$ and $\eta = 0.7$) along mask benefit to others, ${q}_{M}/q$ are shown in Fig. 1. The ratio ${q}_{M}/q$ indicates the benefit of mask-wearing to neighboring people around a masked person. As a general tendency, when masks are used less and efficiency is lower, the total percentage of population that will be infected is very high. The use of higher efficiency masks significantly reduces the fraction of infected individuals when 90% of people wear masks. Figure 2(a) shows that the fraction of infected individuals is lower if the mask benefits only the wearer without any benefit to others, i.e., lower ${q}_{M}/q$ and higher efficiency. Figure 2(b) shows that the fraction of infected individuals considerably decreases when $q = 0.1$, i.e., the ratio of asymptomatic to symptomatic diffusing risk is low.

**Figure 3:**
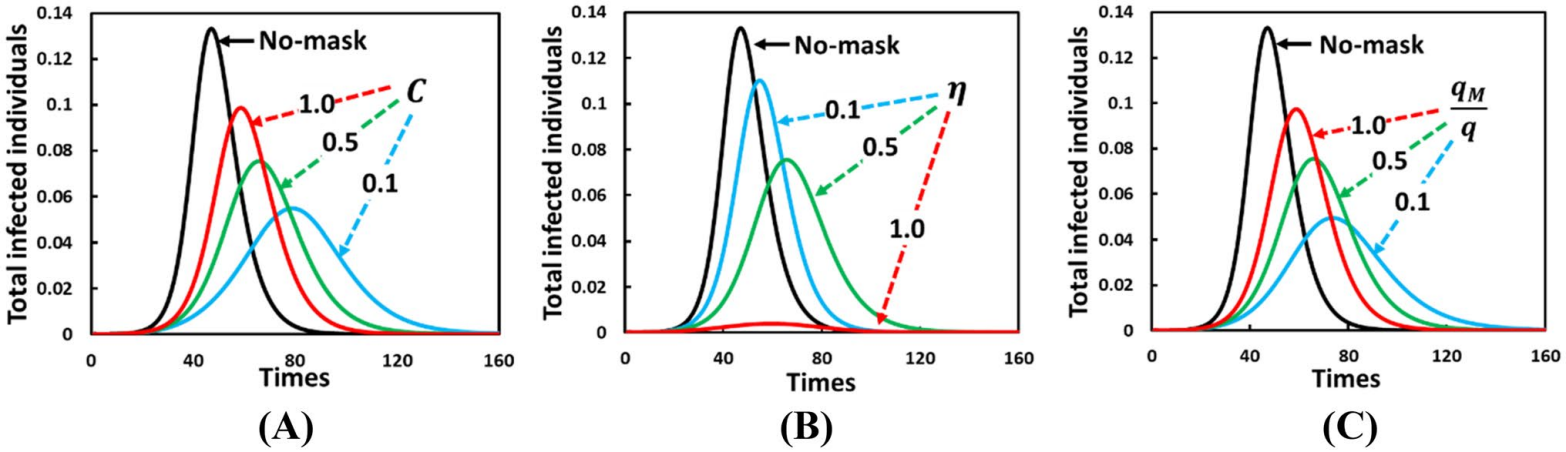
Presented is the total infected individuals as a function of time evolutionary line graph of three different situations with and without (solid black line; no-mask) behavioral dynamics for (A) varying mask cost $\left( C \right)$, (B) varying mask efficiency $\left( \eta  \right)$, and (C) varying mask benefit to others $\left( {q_{M} /q} \right)$. In (A), for settings $C  = 0.1$ (blue), $0.5$ (green), and $1.0~$(red) (also, $\eta = 0.5$, and $q_{M}  = 0.5$), when masks are cheap, individuals are more likely to wear a mask and are less likely to get infected. When mask efficiency is higher (B), parameter $\eta$ control the disease diffusion. Here, $\eta = 0.1$ (blue), $0.5$ (green), $1.0~$(red), and $C = 0.5$, $q_{M} = 0.5.$ Finally, in (C), for settings, $q_{M}  = 0.1$(blue), $0.5$(green), $1.0~$(red), (also, $C = 0.5$, $\eta  = 0.5$), mask benefit to others with lower $q_{M} /q$ have impact to reduce the total infected individuals. Thus, the reduce pick of total infected individuals only when the mask efficiency is high and mask cost is low, and decreasing infected when $q_{M} /q$ is low. Parameters used are,$~q = 1.0$, $q_{C} =  0.1$, $\beta = 0.83333,\gamma = \frac{1}{3},~\alpha  = \frac{1}{6}$, and $\tau  = \frac{1}{4}$.

Figure 3 shows the fraction of total infected individuals over time where (A) shows varying mask costs $(C = 0.1,~0.5,~1.0)$, (B) shows changing mask efficiency $(\eta  = 0.1,~0.5,~1.0)$, and (C) shows varying ratio of mask benefit to others $(q_{M} /q~ = 0.1,~0.5,~1.0)$. In each panel, a solid green line is used for the same settings: $C = 0.5$, $\eta  = 0.5$, and ${q}_{M}/q = 0.5$, whereas the other two colored lines (blue and red) vary depending on focal parameters. Panel A shows that compared with mask cost, the infected fraction can be considerably reduced if a mask is provided at low prices simply because it encourages an individual to use a mask to avoid infection. Meanwhile, panel B shows that even the low efficiency case $(\eta  = 0.1)$ can reduce and delay the peak compared with the case of not wearing a mask (black line). Now, let us observe the sensitivity of ${q}_{M}/q;$ the rate of mask benefit to others (not-wearer) is shown in panel C. We confirmed that mask-wearing benefits others by lowering ${q}_{M}/q$; a more significant peak reduction for all infected individuals can be achieved, which results in a larger time delay. Thus, besides cost, it is worth highlighting how wearing a mask is beneficial to others and directly benefits a wearer by controlling an outbreak.

**Figure 4:**
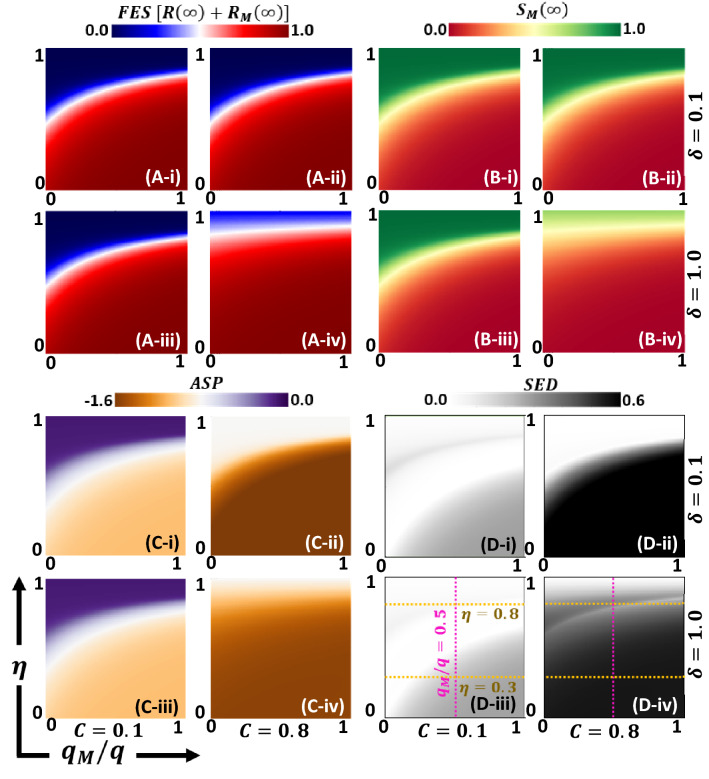
Shown is the (A) Final epidemic size, FES $$\left( {R\left( \infty  \right) + R_{M} \left( \infty  \right)} \right)$$, (B) mask-wearer, $$S_{M} \left( \infty  \right)$$, (C) average social payoff, ASP, and (D) social efficiency deficit, SED as a function of mask efficiency (benefit to wearer), $$\eta$$ and benefit to neighbors around a masked wearer, $${\raise0.7ex\hbox{${q_{M} }$} \!\mathord{\left/ {\vphantom {{q_{M} } q}}\right.\kern-\nulldelimiterspace} \!\lower0.7ex\hbox{$q$}}$$. We present the entire parameter space in four blocks labeled (i), (ii), (iii), and (iv) based on the mask cost (row-wise) and hesitance for mask cost (column-wise) as $$\left( {C = 0.1,~\delta  = 0.1} \right)$$, $$\left( {C = 0.8,~\delta  = 0.1} \right)$$, $$\left( {C = 0.1,\delta  = 1.0} \right)$$, and $$\left( {C = 0.8,\delta  = 1.0} \right)$$. As the mask becomes affordable, a higher effective mask may suppress outbreaks (lower FES) because it achieves higher masked coverage. Also, interesting is that the masked coverage increased when the masked wearing rightly benefitted to neighbors around a masked wearer and sufficiently sensitive to mask cost (lower $$\delta$$). Here, the violet and two yellow dotted lines indicate for $${\raise0.7ex\hbox{${q_{M} }$} \!\mathord{\left/ {\vphantom {{q_{M} } q}}\right.\kern-\nulldelimiterspace} \!\lower0.7ex\hbox{$q$}} = 0.5$$, $$\eta  = 0.3$$ and $$\eta  = 0.8$$, which are shown as line graph in Figs. 4, 5, and 6, respectively . Parameters used are $$q = 1.0,$$ $$q_{C}  = 0.1,$$ and $$\beta = 0.83333,\gamma = \frac{1}{3},~\alpha  = \frac{1}{6}$$, and $$\tau  = \frac{1}{4}$$.

From a holistic perspective, Fig. 4 shows final epidemic size *(FES)* (Panel A), susceptible mask-wearer, $${S}_{M}$$ (Panel B), average social payoff, $$(ASP)$$ (Panel C), and $$SED$$ (Panel D) in the 2D heat map of mask efficiency, $$\eta $$; versus the ratio of mask benefit to others, $${q}_{M}/q $$ (throughout this study, we presumed $$q = 1.0$$); for a different combination of mask cost, $$C;$$ and hesitance for mask cost, $$\delta $$. With an increase in the values of $$\eta $$, the FES is reduced because the number of mask-wearing individuals considerably increases, as expected. People are most likely to wear a face mask when the efficiency (protection capability) is high. Furthermore, with a decrease in mask wearer benefit to others, $${q}_{M} / q$$, FES is decreased ($${S}_{M}$$ increased), which indicates that mask-wearers reduce the spread of disease. With an increase in both mask cost and hesitance [Fig. 4 (A-iv) & (B-iv)], fewer people wear masks owing to higher cost and discomfort, which increases FES.

**Figure 5:**
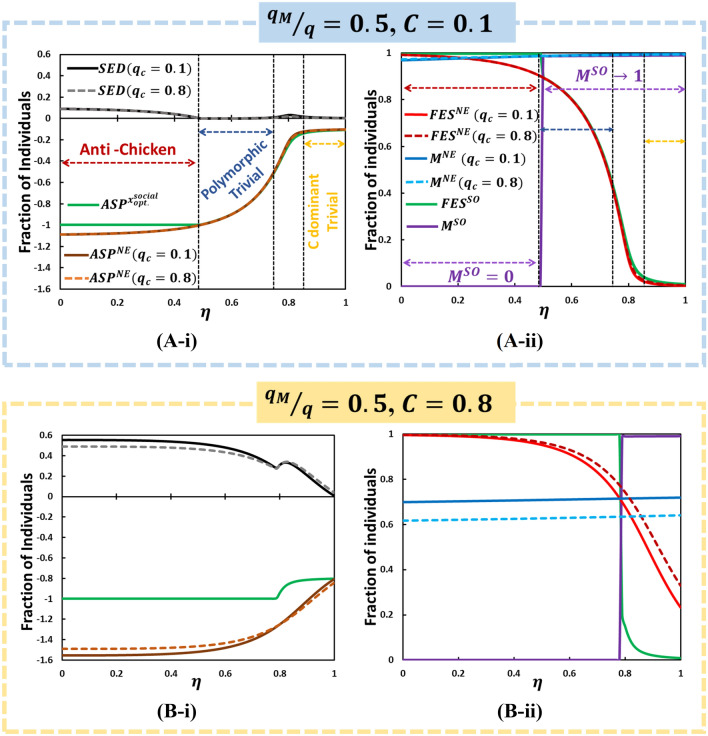
According to Fig. 3(D) for the violet dotted line at fixed , we present line graphs for (*-i) ASP (NE), ASP(SO), SED, (leftmost column) and (ii) FES(NE), FES(SO), M(NE), and M(SO) (rightmost column). In (A-i), with cheaper mask cost shows that the solid black line and the gray dotted line indicate for conformity rates for and , respectively, along with . Also, (A-ii) specify the line graphs for (red), (blue), (green) and (purple). When, , the anti-chicken region has has emerged; dilemma exists and shows Polymorphic characteristics (A-i) and (purple) shows almost perpendicular (a bit sloped) for ; when is less than , mask-wearer social optimum is 0, and in the region , stays at 1 (A-ii). Besides, we observed two dilemma-free regions: polymorphic trivial and C-dominant trivial, having no SED (no dilemma, ) in (A-i). That yielding identical ASP as observed at SO and NE (orange and green), that is, cannot improve the payoff at NE anymore, and, possessing no social dilemma at all. This region's counterpart in FES and mask-wearer (A-ii) is illustrated identical FES for both SO and NE as polymorphic trivial region. The remaining region in (A-i) possesses certain SED levels that depict the presence of a social dilemma that we can define as a transition state between polymorphic trivial and C-dominant trivial. Other parameters used are, $q = 1.0,$ $q_{C}  = 0.1,$ and $\beta = 0.83333,\gamma = \frac{1}{3}, \delta = 1.0, ~\alpha  = \frac{1}{6}$, and $\tau  = \frac{1}{4}$.

Based on Eq. (15), the SED is shown in Fig. 4 (D-*). If an ASP observed at NE is less than that at SO, a certain amount of social dilemma occurs (displayed by gray to black; not whiteout). Therefore, we visually show either an increase in the ratio of mask-wearing benefit to others, $${q}_{M}/q $$, or a decrease in the mask efficiency, $$\eta $$, under higher mask cost and higher hesitancy, which makes an individual suffer from a higher social dilemma of whether to wear a mask as a protecting provision. SED generalizes dilemma strength by measuring the potential for bettering society. If the SED is vast (deep black), there is more room for improvement. In addition, when SED is small, there is little room for improvement through cooperation, which indicates a lower incentive for an individual to wear a mask. The whiteout region shows that no dilemma ensues; society reached its social optimal situation. Figure 5 (D-*) shows that the mask efficiency (which represents the benefit to a wearer and ranges from 0 to 1) works more effectively than $${q}_{M}/q $$ (which represents the benefit to others around a wearer and ranges from 0 to 1), which simplifies this social dilemma structure. This is discussed in detail below.

**Figure 6:**
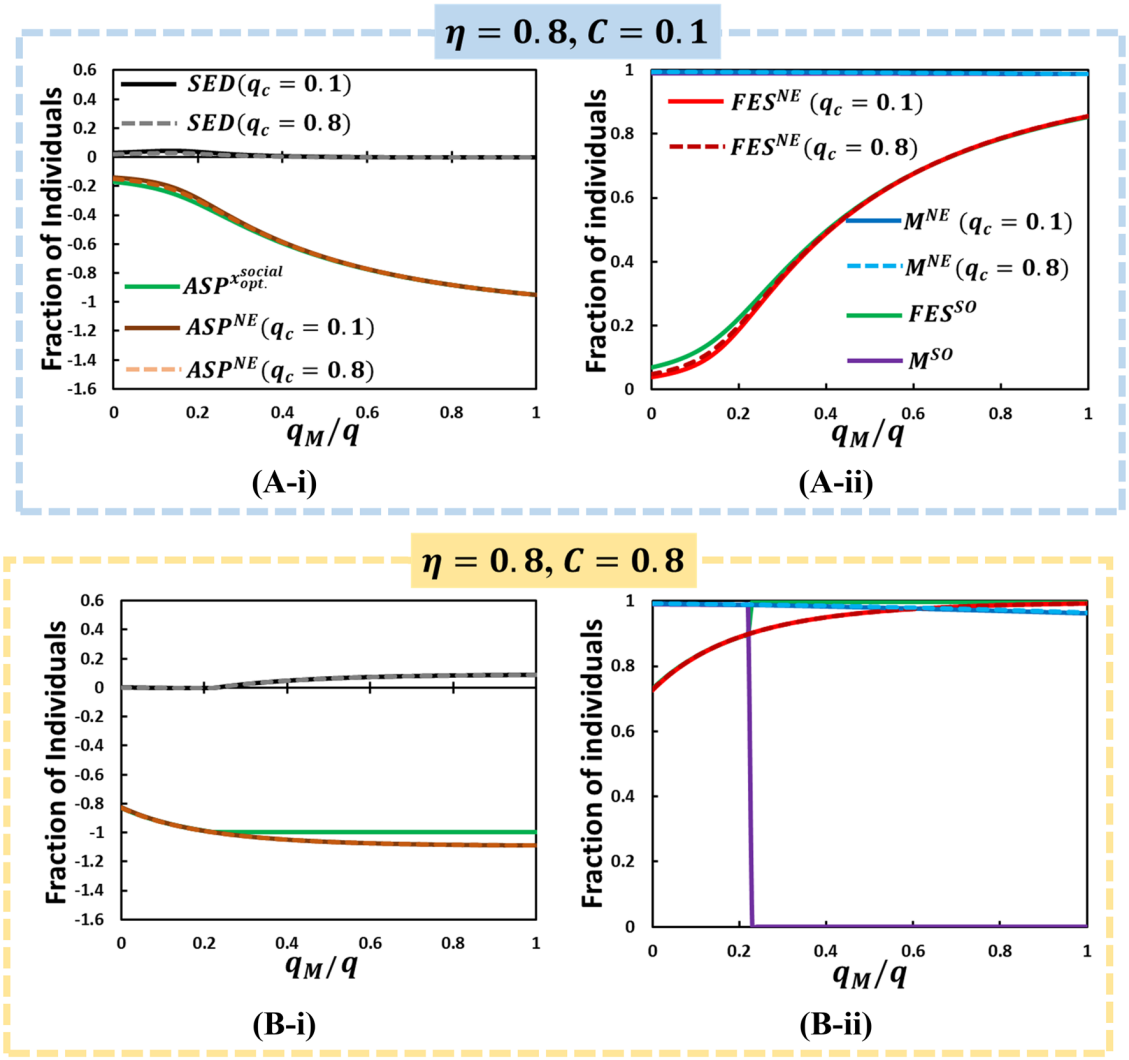
According to Fig. 3(D) for the orange dotted line at fixed , we present line graphs for (i) ASP (NE), ASP(SO), SED, (leftmost column) and (ii) FES(NE), FES(SO), M(NE), and M(SO) (rightmost column) along . In the low cost with high efficiency case (A), SED towards its lowest value (zero) compared to other matters (almost no dilemma situation). It is because wearing a mask encourages people to take the mask, M reaches its peak-point. In (B), although FES and M's holistic tendency except for observes analogous along with what we have mentioned earlier, the mask-wearer for SO (, purple) shows extreme binary (two-fold) situation. Other parameters used are, $q = 1.0,$ $q_{C}  = 0.1,$ and $\beta = 0.83333,\gamma = \frac{1}{3}, \delta = 1.0, ~\alpha  = \frac{1}{6}$, and $\tau  = \frac{1}{4}$.

**Figure 7:**
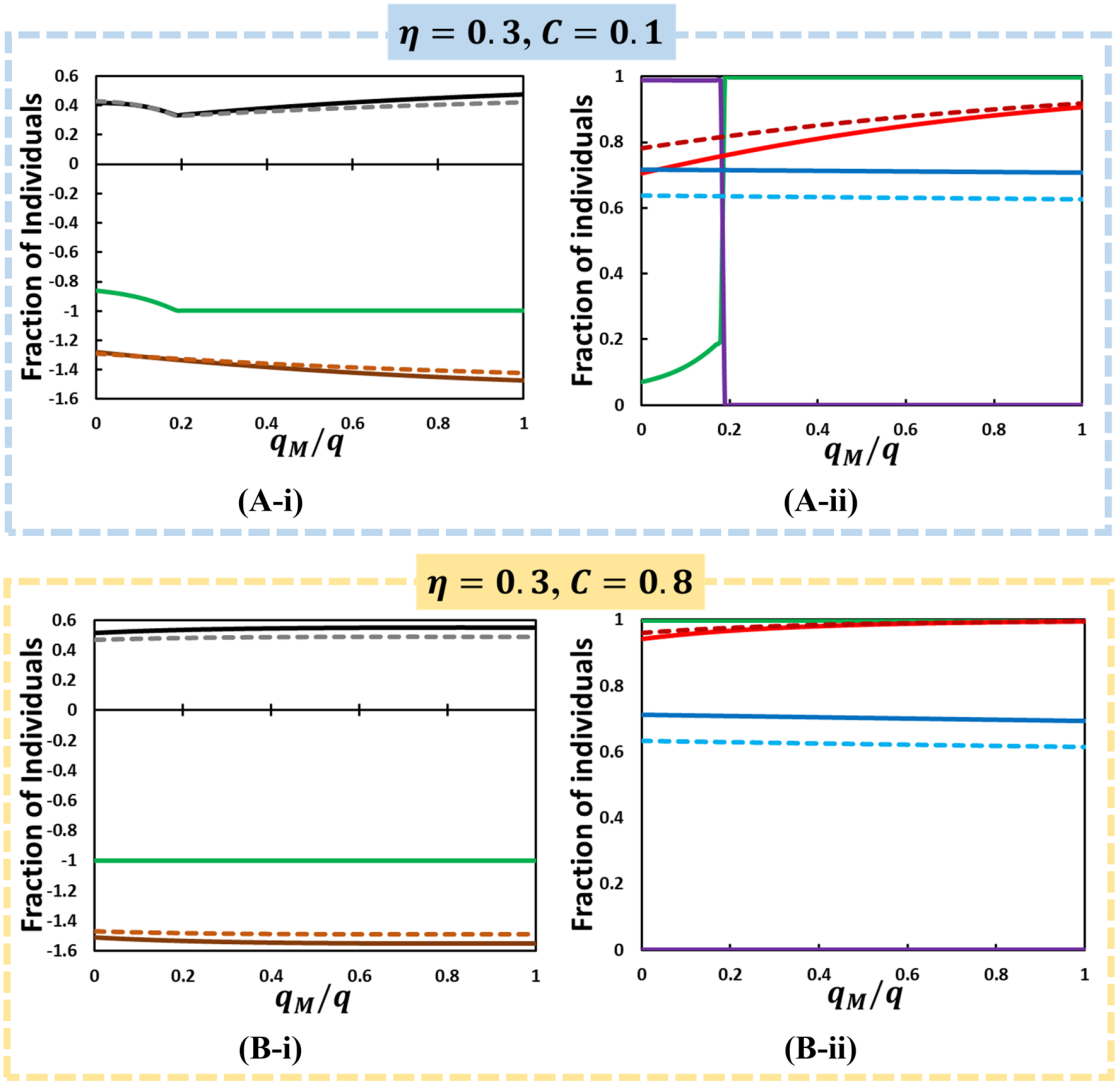
According to Fig. 3(D) for the orange dotted line at fixed lower mask efficiency , we present line graphs for (i) ASP (NE), ASP(SO), SED, (leftmost column) and (ii) FES(NE), FES(SO), M(NE), and M(SO) (rightmost column) along . We also assume two types of conformity effect by varying conformity rate (solid line) and (dotted line) and varying mask cost (A) and (B) . Other parameters, $q = 1.0,$ $q_{C}  = 0.1,$ and $\beta = 0.83333,\gamma = \frac{1}{3}, \delta = 1.0, ~\alpha  = \frac{1}{6}$, and $\tau  = \frac{1}{4}$.

The heat map results [Fig. 3(D)] confirm that $$SED$$, which quantifies the social dilemma region, will be reflected by the epidemic dynamics with EGT in the current model. Now, we evaluate how the mask efficiency and benefit behave with the different parameter perspectives on behavioral dynamics in terms of social disability. These phenomena can be explained using the line graphs in Figs. 5, 6, 7 that reveal how *SED*, *ASP*, *FES*, and *M* $$\left( { = S_{M} \left( \infty  \right) + R_{M} \left( \infty  \right)} \right)$$ at SO and NE (with different mask costs and hesitance levels) vary as a function of $$\eta$$ (Fig, 5) and $${\raise0.7ex\hbox{${q_{M} }$} \!\mathord{\left/ {\vphantom {{q_{M} } q}}\right.\kern-\nulldelimiterspace} \!\lower0.7ex\hbox{$q$}}$$ (Figs. 6, 7).

The most important feature of the current model is that the benefit to the mask wearer and surrounding people can be separately formulated. The former is controlled by $$\eta$$ (larger is more beneficial), whereas the latter is controlled by $${\raise0.7ex\hbox{${q_{M} }$} \!\mathord{\left/ {\vphantom {{q_{M} } q}}\right.\kern-\nulldelimiterspace} \!\lower0.7ex\hbox{$q$}}$$ (smaller is more beneficial). Thus, Fig. 4 (i.e., a set of heat maps of 2D plane of $${\raise0.7ex\hbox{${q_{M} }$} \!\mathord{\left/ {\vphantom {{q_{M} } q}}\right.\kern-\nulldelimiterspace} \!\lower0.7ex\hbox{$q$}}$$ and $$\eta$$) is eloquent and allows us to understand the social dilemma behind people’s decision of whether to wear a mask. However, we need to better understand such heat maps. Therefore, we drew line graphs at different three cross-sections along $${\raise0.7ex\hbox{${q_{M} }$} \!\mathord{\left/ {\vphantom {{q_{M} } q}}\right.\kern-\nulldelimiterspace} \!\lower0.7ex\hbox{$q$}} = 0.5$$ (violet vertical dotted line in Fig. 4 (D-iii) & (D-iv)), $$\eta  = 0.3$$ and $$\eta  = 0.8$$ (yellow horizontal line), which are shown in Figs. 5, 6, 7, respectively.

Figure 5 shows *SED* at two conformity pressure weight factors; $$q_{c}  = 0.1$$ and 0.8 along $$\eta$$, which is accompanied by *ASP* observed at NE;$$~ASP^{{NE}}$$, (for both cases of $$~q_{c}$$) as well as at SO; $$ASP^{{x_{{opt}}^{{social}} }}$$ [see Eq. (15)] in panel (*-i), and *FES*, the total fraction of mask-wearing individuals; $$S_{M} \left( \infty  \right) + R_{M} \left( \infty  \right) \equiv M$$ in panel (*-ii) (observed at NE with varying $$q_{c}$$ and at SO). Panels (A-*) and (B-*) mutually compare the effect of mask cost. Figures 6 and 7 are presented in the abovementioned format, except for the X-axis, which is $${\raise0.7ex\hbox{${q_{M} }$} \!\mathord{\left/ {\vphantom {{q_{M} } q}}\right.\kern-\nulldelimiterspace} \!\lower0.7ex\hbox{$q$}}$$.

Let us start with Fig. 5 (A-*). In the higher $\eta$ [> 0.83 (approx.)] region, owing to the absence of a gap in ASP at NE and SO, there is no social dilemma (*SED*  =  0). It is realized by $M^{{NE}}$ = 1, which successfully suppresses FES and is quite low. The value is the same irrespective of either higher or lower $q_{c}$. Referring to the knowledge of EGT^57–61^, such a situation can be described as the “cooperation (C) dominant Trivial” game.

In the middle range of $\eta$ [0.49 < $\eta$ < 0.74(appr.)], another non-dilemma phase appears that is confirmed by zero SED. Unlike previous situations, it can be realized by a slightly lower $M^{{NE}}$(< 1), which results in a reasonably larger FES because of mask efficiency, which indexes the benefit of wearing a mask, is not as high as the C dominant Trivial phase. Such a relatively lower mask efficiency and nonzero cost, which occurs SO, is consistent with what can be observed at NE because allowing disease spreading to some extent owing to mask’s incapability allows to realize the optimal social situation. This specific situation is called “Polymorphic Trivial” because it results in $M^{{NE}}  < 1$ (mask-wearing and non-mask wearing groups coexist); however, NE is still consistent with the optimal social situation.

Of note, between C dominant Trivial and Polymorphic Trivial, nonzero SED, i.e., a social dilemma emerges. Nevertheless, its extent is subtle because of the transitional phase between both Trivial game structures.

At lower $\eta$ [$\eta$ < 0.49 (appr.)], another social dilemma differs from the above mentioned one and for which the gap of ASP is larger. Nonzero SED implies that an evolutionary equilibrium can be improved to $ASP^{{x_{{opt}}^{{social}} }}$. Unlike the conventional story that is commonly observed in real social dilemma structures, what can be observed in this particular phase is quite ironic and substantially interesting in terms of EGT. In the focal region, the fact of $ASP^{{x_{{opt}}^{{social}} }}$ > $ASP^{{NE}}$ resulting from $FES^{{SO}} \left( { = 1} \right) > FES^{{NE}}$ owing to the quite large gap of $M^{{SO}} \left( { = 0} \right) < M^{{NE}}$ implies that none of the people wearing mask and allowing a full-scale epidemic is SO because mask efficiency is quite low, and the cost is nonzero. Yet, the evolutionary process backed by the behavioral dynamics that we assumed makes many people willing to wear a mask even if almost nothing minimizes the social cost, which implies that the risk of infection is overestimated by an individual, which results from the balance of $\left( {I^{S}  + I_{M}^{S} } \right)$ in Eq. (12) with other remaining terms in the brackets. This dilemma extent increases when presuming higher mask cost; $C = 0.8$, shown in panel (B-*). Again, by referring to EGT, let us call this social dilemma structure, “Anti-Chicken,” where NE suggests coexisting mask-wearing and non-wearing individuals. However, SO can appear at a non-wearing state, unlike the usual (pure-) Chicken game with the coexistence of cooperative and defective strategies and optimal social situation appearing at all-cooperators-state.

Let us evaluate Figs. 6 and 7. In Fig. 6 (A-*), owing to high mask efficiency and low mask cost, a dilemma-free situation is realized, except the lower region of $${\raise0.7ex\hbox{${q_{M} }$} \!\mathord{\left/ {\vphantom {{q_{M} } q}}\right.\kern-\nulldelimiterspace} \!\lower0.7ex\hbox{$q$}}$$. The lower region of $${\raise0.7ex\hbox{${q_{M} }$} \!\mathord{\left/ {\vphantom {{q_{M} } q}}\right.\kern-\nulldelimiterspace} \!\lower0.7ex\hbox{$q$}}$$ has a slightly small SED, which originates from $$FES^{{SO}}$$ that is slightly larger than $$FES^{{NE}}$$, which results from a small difference between $$M^{{SO}}$$ and $$M^{{NE}}$$($$M^{{SO}}  < M^{{NE}}$$). If the mask cost considerably increases [Fig. 6 (B-*)], such tendency is significantly altered. In the region of lower $${\raise0.7ex\hbox{${q_{M} }$} \!\mathord{\left/ {\vphantom {{q_{M} } q}}\right.\kern-\nulldelimiterspace} \!\lower0.7ex\hbox{$q$}}$$ [$${\raise0.7ex\hbox{${q_{M} }$} \!\mathord{\left/ {\vphantom {{q_{M} } q}}\right.\kern-\nulldelimiterspace} \!\lower0.7ex\hbox{$q$}}$$ < 0.24(appr.)], C dominant Trivial phase appears ($$M^{{SO}}  = 1$$ and $$M^{{NE}}  = 1$$; irrespective of $$~q_{c}$$). Beyond the threshold of $${\raise0.7ex\hbox{${q_{M} }$} \!\mathord{\left/ {\vphantom {{q_{M} } q}}\right.\kern-\nulldelimiterspace} \!\lower0.7ex\hbox{$q$}}$$, $$M^{{SO}}$$ becomes zero, whereas $$M^{{NE}}$$ remains approximately 1, irrespective of $$~q_{c}$$. According to the abovementioned terminology of “Anti-Chicken,” this specific social dilemma should be called “Anti-Prisoner’s Dilemma (PD).” The occurrence of such social irony is related to how wearing a mask is beneficial to other people than to a wearer, which is quantified by $${\raise0.7ex\hbox{${q_{M} }$} \!\mathord{\left/ {\vphantom {{q_{M} } q}}\right.\kern-\nulldelimiterspace} \!\lower0.7ex\hbox{$q$}}$$. If the case is highly beneficial to others (lower $${\raise0.7ex\hbox{${q_{M} }$} \!\mathord{\left/ {\vphantom {{q_{M} } q}}\right.\kern-\nulldelimiterspace} \!\lower0.7ex\hbox{$q$}}$$), wearing mask is fully justified from the SO standpoint ($$M^{{SO}}  = 1$$), which is fairly followed by the evolutionary process ($$M^{{NE}}  = 1$$). In contrast, beyond the threshold, although the SO suggests abolishing the mask, the evolutionary process is still absorbed with an exceptionally high mask-wearing fraction. In the wake of COVID-19, one of the overwhelming reactions observed in the USA^62,63^ is the social controversy of whether one should wear a mask and to obey the request from the public health authority cooperatively. One of the opinions supporting “not” is that whether wearing a mask or not is the subject of an individual’s liberties. Interestingly, what has been observed in Japan is entirely opposite. Such a gap between specific two countries has been explained by social compliance among people. If there are people who underestimate (or overestimate) the risk of COVID-19, question the benefits of wearing a mask to himself and others around him, and instead address “freedom” than “social conformity,” which is fully modeled in the brackets of Eq. (12), they may behave relatively close to what the optimal social situation shows than what the NE shows.

The phase change observed at $M^{{SO}}$ from 0 (1) to 1 (0), as confirmed in Fig. 6 (B-ii), appears even at lower mask efficiency and lower cost [Fig. 7 (A-ii)].

**Figure 8:**
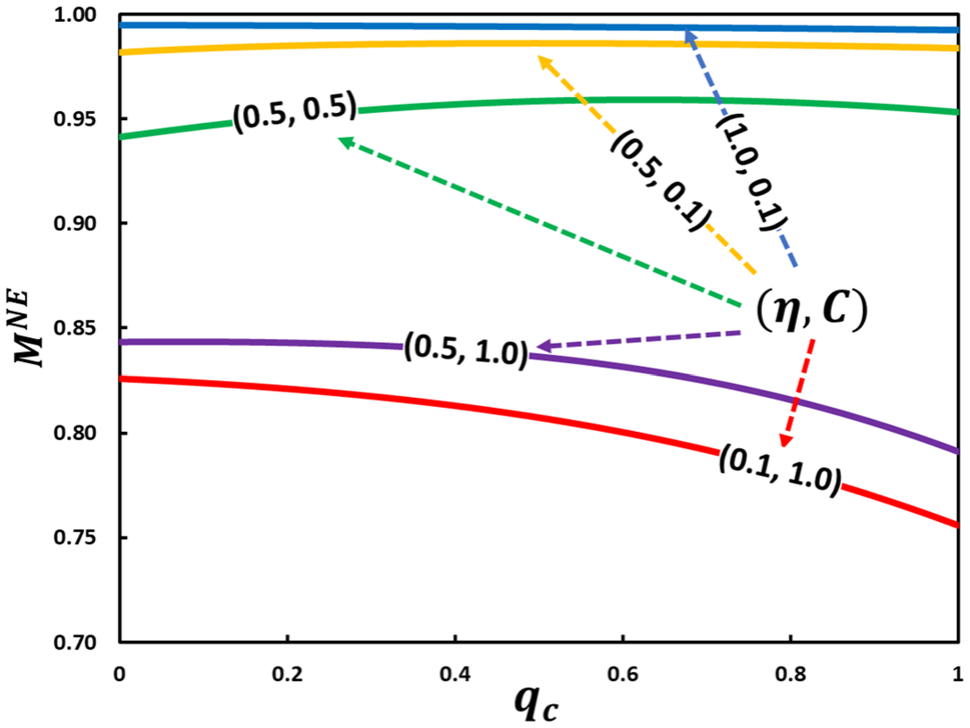
Shown the effects of conformity adoption on the fraction of mask-wearing individuals, as a function of the weight factor owing to conformity pressure rate, for various mask cost and its efficiency. While constructing the figure, we take for intermediate plan (green), and for two extreme plans, higher efficiency with lower cost (blue) and vice versa (red), respectively. Also, the lines colored with solid orange and purple are drawn for the settings of and The baseline values for the parameters , , and are considered. See the accompanying explanation in the main text for details.

In Fig. 8 is a line graph for mask-wearing individuals at equilibrium, $M^{{NE}}$, along with the conformity rate, $q_{c}$, by varying mask efficiency and cost. If the mask cost is low, $C = 0.1$ (blue and orange), the mask-wearer fraction is relatively high $\left( {M^{{NE}}  \to 1} \right)$, irrespective of the conformity effect $q_{c}$, because the price does not convey any burden to an individual. At intermediate settings for cost and efficiency at (0.5, 0.5) (green), higher $M^{{NE}}$ and lower sensitivity to the conformity effect are still observed. However, people are not interested in wearing masks owing to higher costs $\left( {C = 1.0} \right)$, which reduces the fraction of mask-wearers (purple and red). The sensitivity from the weight factor owing to conformity pressure, $q_{c}$, at $C = 1.0$, shows that the entire mask-wearing fraction is monotonically decreasing with $q_{c}$, which is also conceivable. By deliberately observing all lines, we noted an interesting propensity that when a cheaper mask, $C = 0.1$ and $C = 0.5$, is available, with an increase in $q_{c}$, first, $M^{{NE}}$ increases and reaches its peak and then decreases even though it is not significant. Thus, social conformity pressure works both ways. This is called the bandwagon effect, which compels people to wear a mask due to it being in vogue as opposed to refusing a mask due to prevailing attitudes.

## Discussion

In the COVID-19 pandemic, there is an ongoing debate on whether to endorse wearing face masks to reduce the spread of infection^64^. However, most health experts recommend mask use, which addresses the general preventive principle when the baseline risk is very high, and none of the established medical treatments, such as a vaccine and antiviral drugs, are available. An important aspect is that mask-wearing provides not only a certain extent of benefit to a wearer but also to others around him. Nevertheless, mask-wearing can feel cumbersome, unpleasant, and costly, which may be more important. Therefore, mask-wearing poses a similar structure of the “vaccination dilemma.” In each scenario, individuals benefit form actions (mask wearing and vaccination, respectively) that yield benefits to the group, but which are personally costly. Based on modeling, the effect of mask-wearing should be divided into the benefit to a wearer and to others in an explicit formulation.

The model reported here deals with this point, where epidemic dynamics based on the SEIR process and behavior dynamics are deliberately quantified. Numerical results successfully highlight the focal point, as mentioned above. The benefit to a wearer dominates the benefit to other people, which emerges as a social dilemma. The structure can be quite diverse and rich and contain a variant of PD, Chicken-type dilemma, and anti-Chicken dilemma in addition to the Trivial game structure.

In conclusion, this study contains the possibility of comparative and absolute advantages of wearing a mask originating from mask-wearers. The abovementioned results show the feasibility of analyzing united multifaceted epidemics and EGT of the pandemic situation. We expect that such an outline should influence policymakers’ endorsements, starting with the relevant stakeholders’ involvement with further progress.

## Methods

To appropriately show the disease process (Fig. 1A) observed during the COVID-19 pandemic, there are two important factor to consider: i) implementation of the exposed period in which an infected individual is not infectious and ii) infectious but has a mild symptom state (called asymptomatic infected), which should be distinguished from the usual infected stage (called symptomatic infected). Hence, we considered a population consisting of susceptible $(S)$, exposed $(E)$, asymptomatic infected $({I}^{A})$, symptomatic infected $({I}^{S})$, and recovered $(R)$ who did not wear a mask. Within mask-wearing people, we further divided the population into mask-wearing groups, i.e., susceptible $({S}_{M})$, exposed $({E}_{M})$, asymptomatic infected $({I}_{M}^{A})$, symptomatic infected $({I}_{M}^{S})$, and recovered $({R}_{M})$. The system of differential equations governing this mask-wearing epidemiological model is as follows:

1 $$ \frac{{dS}}{{dt}} =  - \beta  \cdot S\left( {I^{S}  + I_{M}^{S}  + q \cdot I^{A}  + q_{M}  \cdot I_{M}^{A} } \right) - x_{M}  \cdot S, $$

2 $$ \frac{{dS_{M} }}{{dt}} = x_{M}  \cdot S - \beta \left( {1 - \eta } \right)S_{M} \left( {I^{S}  + I_{M}^{S}  + q \cdot I^{A}  + q_{M}  \cdot I_{M}^{A} } \right), $$

3 $$ \frac{{dE}}{{dt}} = \beta  \cdot S\left( {I^{S}  + I_{M}^{S}  + q \cdot I^{A}  + q_{M}  \cdot I_{M}^{A} } \right) - \alpha  \cdot E,  $$

4 $$ \frac{{dE_{M} }}{{dt}} = \beta \left( {1 - \eta } \right)S_{M} \left( {I^{S}  + I_{M}^{S}  + q \cdot I^{A}  + q_{M}  \cdot I_{M}^{A} } \right) - \alpha  \cdot E_{M} ,  $$

5 $$ \frac{{dI^{A} }}{{dt}} = \alpha  \cdot E - \tau  \cdot I^{A} , $$

6 $$  \frac{{dI_{M}^{A} }}{{dt}} = \alpha  \cdot E_{M}  - \tau  \cdot I_{M}^{A} ,  $$

7 $$ \frac{{dI^{S} }}{{dt}} = \tau  \cdot I^{A}  - \gamma  \cdot I^{S} , $$

8 $$ \frac{{dI_{M}^{S} }}{{dt}} = \tau  \cdot I_{M}^{A}  - \gamma  \cdot I_{M}^{S} , $$

9 $$  \frac{{dR}}{{dt}} = \gamma  \cdot I^{S} ,  $$

10 $$  \frac{{dR_{M} }}{{dt}} = \gamma  \cdot I_{M}^{S} ,   $$

where ${x}_{M}$ is the wearing mask rate at which the fraction of $S$ individuals convert to ${S}_{M}$, and it is governed by the behavioral dynamics in EGT. The behavioral components ${x}_{M}$ will be increased or decreased depend on expected payoff differences that allow only the fraction of suspected susceptible individuals can choose either wearing a mask or not. An infected individual, however, is not permitted to change behavior from non-masked to masked wearing person. Herein, only one connection between susceptible (non-masked) to mask-wearing susceptible is considered to avoid unnecessary complexity^18^. Regarding the epidemic dynamics, $\beta$,$\alpha$, $\tau$, and $\gamma$ are disease transmission, incubation, asymptomatic to symptomatic infected, and recovery rates, respectively (see Table 1). Parameter $\eta$ is the mask efficiency, which is defined by the concept of the “efficiency model”^56^, which directly shows the mask’s filtering capability, i.e., the mask’s ability to protect the wearer from infectious particles. For example, an N95 mask is intended to block $95\% ~(\eta  = 0.95)$ of tiny 0.3-µm particles. The parameter $q$ represents the rate of spreading the virus from an asymptomatic individual who is not wearing a mask compared with a symptomatic one. In contrast, ${q}_{M}$ represents the case when an asymptomatic individual wears a mask. Let us call both $q$ and ${q}_{M}$ be the “rate of asymptomatic to symptomatic diffusing risk” when not-wearing and wearing a mask, respectively. We assumed $1>q>{q}_{M}$. In a nutshell, ${q}_{M}/q$ quantifies the benefit of mask-wearing to surrounding people around a focal individual who wears a mask, whereas $\eta$ indicates the direct benefit of mask-wearing to the individual (Fig. 1B and c). An important point to be confirmed in our assumption is that an individual in the state of ${I}_{M}^{A}$ does wear a mask; for the state of symptomatic individuals (${I}_{M}^{S}$), wearing a mask or not does not reduce the risk to others.

**Table 1:** Parameters used in this work with descriptions.

Symbols	Descriptions
$\beta$	The disease transmission rate
$q$	Rate of asymptomatic to symptomatic diffusing risk for not-wearing mask
$q_{M}$	Rate of asymptomatic to symptomatic diffusing risk for wearing mask
$q_{M} /q$	Benefit of mask-wearing to neighboring people
$x_{M}$	Wearing mask rate
$\eta$	Mask efficiency
$\alpha$	Incubation period rate
$\tau$	Asymptomatic to symptomatic infected rate
$\gamma$	Recovery rates
$m$	Proportionality constant
$\delta$	Sensitivity/hesitance of mask cost
$C_{M}$	Perceived mask cost
$C_{I}$	Infection cost
$C$	Relative mask cost, $C = C_{M} /C_{I} ,$ $\left( {C_{I} = 1.0} \right)$
$q_{c}$	Weight factor owing to conformity pressure

We impose the following constraint:

11 $$  S\left( t \right) + E\left( t \right) + I^{A} \left( t \right) + I^{S} \left( t \right) + R\left( t \right) + S_{M} \left( t \right) + E_{M} \left( t \right) + I_{M}^{A} \left( t \right) + I_{M}^{S} \left( t \right) + R_{M} \left( t \right) = 1. $$

### Behavioral dynamics

Applying the human behavioral dynamics to the EGT concept, individuals change their strategy adoption owing to the perceived risk of infection, cost, (Fig. 1D) and conformity effect. A cooperator (self-consuming mask wearer) expects to suffer a perceived cost, ${C}_{M}$, with the sensitivity/hesitance of mask cost $\delta$. A defector (non-wearing individuals) has a perceived risk based on the fraction of total symptomatic infected individuals $({I}^{S}+{I}_{M}^{S})$ multiplied by the perceived disease cost ${C}_{I}$. In the following, we normalized ${C}_{M}$ with $C$ presuming ${C}_{I} = 1.0$. Thus, the payoff gain depends on the difference between the perceived payoff of a mask wearer $\left[-\delta \cdot C\right]$ and the payoff for risking infection $\left[-({I}^{S}+{I}_{M}^{S})\times 1.0\right]$. To obtain the conformity effect^42^ (i.e., social pressure amid wearing a mask or not wearing it), we assume $\frac{\left({M}^{*}-W{M}^{*}\right)}{{M}^{*}+W{M}^{*}} = ({M}^{*}-W{M}^{*})$. Here ${M}^{*}$ and ${WM}^{*}$ represent the total number of individuals who wear and do not wear a mask, respectively. Parameter ${q}_{c}$ is the weight factor owing to conformity pressure and $m$ is the proportionality constant. The expected payoff gains for altering strategies can be measured as $({I}^{S}+{I}_{M}^{S})-\delta C+{q}_{c}({M}^{*}-W{M}^{*})$, which can be expressed (imitation dynamics) for the time evolution of ${x}_{M}$ as follows:

12 $$\frac{d{x}_{M}}{dt} = m{\cdot x}_{M}\left[1-{x}_{M}\right]\left[({I}^{S}+{I}_{M}^{S})-\delta \cdot C+{q}_{c}({M}^{*}-W{M}^{*})\right],$$

where,

13.1 $${M}^{*}(t) = {S}_{M}\left(t\right)+{E}_{M}\left(t\right)+{I}_{M}^{A}\left(t\right)+{I}_{M}^{S}\left(t\right)+{R}_{M}\left(t\right),$$

13.2 $$ WM^{*} (t) = S(t) + E(t) + I^{A} (t) + I^{S} (t) + R(t). $$

Here, Eq. (12) represents the behavioral dynamics in which the “third brackets” give the internal equilibrium for ${x}_{M}$ other than two trivial equilibria at ${x}_{M} = 0$ and $1$^5,10,26,41,55^. The general framework of the individual behavior, which allows an individual to change decision depends mainly on the “third bracket”; either increasing ${x}_{M}$ (positive) or decreasing (negative). The first term in the “third brackets,” the total number of visible infected people works to drive individuals compliant to wear a mask. Let us keep the first term as a reference; the mask cost in the second term acts to let them hesitate to take the mask. Yet, the sensitivity of the second term to the first term should be noted because the infected fraction has a different physical dimension from that of cost. Thus, we need to introduce another parameter, $\delta$, which implies the sensitivity of mask cost to the influence of infected fraction. Finally, the last term implies the conformity effect by referring to $({M}^{*}-W{M}^{*})$, in which the parameter, ${q}_{c}$, accounts for the weight factor to conformity pressure.

### ASP and SED

We evaluated the holistic social efficiency by considering both disease and mask-wearing costs. FES is defined as the sum of recovered individuals [$FES = {R}_{M}\left(\infty \right)+R\left(\infty \right)$]. We estimated the ASP at NE (social equilibrium)for all possible values of mask cost, $C$, and individual fraction at equilibrium $(t\to \infty )$ for $S$, ${S}_{M}$, ${R}_{M}$, and $R$. In addition, we evaluated ASP at SO without a game aspect by considering the maximum ASP for each cost, $C$, which ranges depending on the wearing mask rate, ${x}_{M}$, from 0 to 1. Finally, the SED (social efficiency deficit)^43–45^ is defined as the difference between ASPs at SO and NE. To unveil the existence of a social dilemma associated with evolutionary game systems, “social efficiency deficit” has been introduced to quantify the payoff difference between social optimum (SO) (the desired state of affairs) and Nash equilibrium (NE). So that one can evaluate the SED in any context and hence predict a social dilemma. If SED  =  0 implies no social dilemma, while any social dilemma causes a positive SED.

We can define ASP (NE) as,

14 $$ASP = -C\cdot {S}_{M}\left(\infty \right)-\left(C+1\right){R}_{M}\left(\infty \right)-R\left(\infty \right)$$

Then, we can define SED as follows,

15 $$SED = {ASP}^{{x}_{opt}^{social}}-{ASP}^{NE}.$$

Starting with the initial assumption, $S\left(0\right)\approx 1$, ${I}^{A}\left(0\right)\approx 0$, ${I}_{M}^{A}\left(0\right)\approx 0$, and ${S}_{M}\left(0\right) = E\left(0\right) = {E}_{M}\left(0\right) = {I}^{S}\left(0\right) = {I}_{M}^{S}\left(0\right) = {R}_{M}\left(0\right) = R\left(0\right) = 0$, the specified set of Eqs. (1–15) is numerically solved using the finite difference method. Throughout numerical simulations, we assumed the basic reproduction number $R_{0}  = 2.5~(\beta  = 0.83333,\gamma  = 1/3)$ and $m = 0.1$. For simplicity, we set the initial mask-wearing rate as small as possible, ${x}_{M}\left(0\right) = 0.00001$.
